# Supplementary material for: Quantity and Source of Protein during Complementary Feeding and Infant Growth: Evidence from a Population Facing Double Burden of Malnutrition
Source: Nutrients. 2022 Sep 23;14(19):3948. doi: 10.3390/nu14193948 (PMC9572535; doi:10.3390/nu14193948)
Supplement: Supplementary file 1 [file nutrients-14-03948-s001.zip › nutrients-1878889-supplementary.pdf]

Supplement materials

Figure S1. An example of nutrient values reported from the INMUCAL program.

ID	Sex	Status	PregLact	Age (year)	Age (month	TotalDay	ENER (kcal)	CHO (g)	FAT (g)	PRO (g)	PROA (g)	PROV (g)
CMU001	M	N	0	1	0	3	1178.771	145.2565	47.99177	41.45483	35.1025	6.35233
CMU002	M	N	0	1	0	3	1063.432	160.3275	29.42906	39.3151	17.527	21.7881
CMU003	F	N	0	1	0	3	925.9052	136.0987	26.22781	36.36519	28.51148	7.853709
CMU004	F	N	0	1	0	3	696.0474	78.14611	30.41309	27.43638	22.25091	5.18547
CMU005	F	N	0	1	0	3	411.589	58.6317	12.81562	15.4304	12.35558	3.07347
CMU006	M	N	0	1	0	3	440.1288	56.83593	14.2008	21.24454	17.24828	3.996259
CMU007	M	N	0	1	0	3	564.6176	74.43222	18.64109	24.77977	23.20266	1.577109
CMU008	M	N	0	1	0	3	523.2179	61.66603	22.43118	18.66833	16.28741	2.380452
CMU009	F	N	0	1	0	3	483.9108	62.92223	19.47962	14.22639	11.54997	2.676423
CMU010	M	N	0	1	0	3	581.7678	79.90227	18.70384	23.45611	20.2161	3.24001

CA (mg)	P (mg)	PHYT (mg)	FE (mg)	FEA (mg)	FEV (mg)	ZN (mg)	VITA (RAE)	VITB1 (mg)	VITB2 (mg)	VITC (mg)
1153.395	894.2804	22.62424	12.72488	10.18167	2.290318	6.398373	1116.543	0.276608	0.259284	154.9203
540.4492	284.7039	61.11697	5.361807	1.139051	4.094088	2.277736	26.7976	1.865258	2.005671	25.71338
279.0734	445.2144	18.86472	4.117639	2.255459	1.852671	2.392353	294.7071	0.475597	0.547151	176.2388
237.309	352.9884	5.64667	3.139947	2.075892	0.975915	2.824645	495.5422	0.375925	0.575913	33.20098
96.64411	169.2282	30.7981	2.16006	1.125943	1.032196	1.319863	1114.533	0.267989	0.271758	28.70562
90.96877	208.7295	13.3298	2.700542	2.012201	0.688341	1.754863	2395.158	0.251963	0.609258	28.73577
511.8628	510.0616	0.421993	5.794385	5.267913	0.418199	5.08275	295.3733	0.438105	1.033329	85.35294
236.1745	189.9803	2.234804	2.30733	1.702067	0.604622	1.563532	851.8967	0.326431	0.374808	28.6019
247.2017	232.2722	0.264617	4.017798	3.448442	0.456441	3.013841	1291.344	0.409234	0.685019	74.23082
371.1164	418.7889	16.14278	6.561127	5.81819	0.720601	3.796531	345.3231	0.486939	0.863801	88.25739
		13.424	13.70277	12.67769	0.312642	6.709003	1005.459	0.864882	1.883625	104.1605

ENER – energy intake (kcal/day); CHO – carbohydrate intake (g/day); PRO – protein intake (g/day); PROA – protein intake from animal source foods (g/day); PROV – protein intake from plant-based foods (g/day); CA – calcium intake (mg/day); P – phosphorus intake (mg/ day); FE – iron intake (mg/day); FEA – iron intake from animal source foods (mg/day); FEV – iron intake from plant-based foods (mg/day); ZN – zinc intake (mg/day); VITA – vitamin A intake (retinal activity equivalent/day); VITB1 – vitamin B1 intake (mg/day); VITB2 – vitamin B2 intake (mg/day); VITC – vitamin C intake (mg/day)

Figure S2. A participant flow chart.

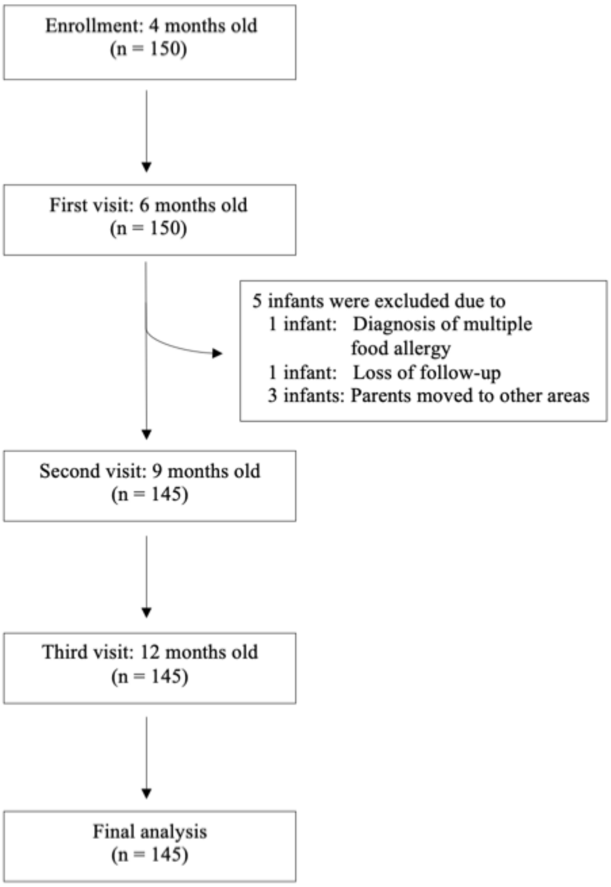

Table S1. Means, standard deviation (SD) and 95% confidence intervals (CI) of conditional growth among protein intake groups.

Conditional	**High intake** (n = 36)		**Median intake** (n = 73)		**Low intake** (n = 36)	
	**Mean (SD)**	**95% CI**	**Mean (SD)**	**95% CI**	**Mean (SD)**	**95% CI**
**WAZ**	0.54 (0.86)	0.25, 0.83	-0.13 (0.95)	-0.35, 0.09	-0.28 (1.04)	-0.63, 0.07
**WLZ**	0.58 (0.99)	0.25, 0.91	-0.14 (0.95)	-0.36, 0.08	-0.30 (0.88)	-0.59, 0.003
**BMIZ**	0.46 (1.08)	0.10, 0.83	-0.13 (0.94)	-0.35, 0.09	-0.27 (0.84)	-0.56, 0.01
**LAZ**	0.07 (1.18)	-0.33, 0.47	-0.01 (0.82)	-0.20, 0.18	-0.04 (1.14)	-0.42, 0.35

Table S2. Complementary feeding recommendations for Thai infants.

**Age** / **Meal composition**	**6 M**	**7M**	**8M**	**9-12M**
Number of main meals	1	1	2	3
Composition of main meal (daily)				
Carbohydrate	finely mashed rice 2 tbs	soft, cooked rice 3 tbs	soft, cooked rice 8 tbs	soft, cooked rice 12 tbs
Protein	½ egg yolk or other meats 1 tbs	½ egg or other meats 1 tbs	1 egg or other meats 2 tbs	1.5 egg or other meats 3 tbs
Vegetables	cooked vegetable ½ tbs	cooked vegetable 1 tbs	cooked vegetable 2 tbs	cooked vegetable 4.5 tbs
Fruits	ripe fruit 1 piece	ripe fruit 2 pieces	ripe fruit 3 pieces	ripe fruit 4 pieces
Added vegetable oils	2.5 ml	2.5 ml	2.5 ml	2.5 ml

M – months old; tbs – tablespoon

Table S3. Comparison of average IGF-1, IGFBP-3 and insulin concentrations between female and male infants at 12 months of age.

Results		All infants (n = 145)	Male (n = 74)	Female (n = 71)
**IGF-1 (ng/ml)**	Mean (SD)	53.87 (26.73)	47.95 (23.73)	59.98 (28.41)
	Mean difference[1] (95% CI)	N/A	-12.03[2] (-20.96, -3.09)	
**IGFBP-3 (ng/ml)**	Mean (SD)	2,649.05 (683.55)	2,500.56 (757.20)	2,808.79 (556.91)
	Mean difference[1] (95% CI)	N/A	-308.22[3] (-531.86, -84.59)	
**Insulin (µU/ml)**	Mean (SD)	7.04 (5.65)	6.85 (5.29)	7.24 (6.02)
	Mean difference[1] (95% CI)	N/A	-0.39[4] (-2.48, 1.71)	

SD – standard deviation; CI – confidence interval; N/A – not analysis; [1]Mean difference and 95%CI were calculated by student's t-test; [2]$p = 0.009$; [3]$p = 0.007$; [4]$p = 0.71$
